# Managing Alzheimer's patients in rural areas of developing country : Experiences of community NGO

**DOI:** 10.1002/alz70860_096312

**Published:** 2025-12-23

**Authors:** S Pal

**Affiliations:** ^1^ SFCCP, meerut, India

## Abstract

**Background:**

National‐health‐registry based data demonstrates Psychosocial support & treatment‐availability are major issues for Alzheimer's ‐sufferers in resource‐poor‐nations. Hence our Non‐Government‐Organisation analysed & made this public health‐policy paper recommendation's.

NGO's are close to rural/tribal‐communities. Cost of running NGO is economical than medical‐institution. Medical treatment cost is prohibitive and inaccessible for majority patients in Asia. Government‐Health‐Departments need to workout strategy to increase access to care.

In resource‐poor‐setting unaffordable cost leads to poor‐therapeutic‐compliance & therefore high mortality. We developed training program to evolve sound /sustainable Alzheimer's‐care for rural communities

**Methods:**

No national‐program for financial help to Alzheimer's patients exists in india. Treatment programs designed towards rural/tribal population needed. Since four years we offer guidance for drug‐therapy‐funding. This project is unique, as we are training NGO health workers to assist patients community in improving access to care. Depending on support given by donors, we provide financial‐assistance for treatment. We improved access to governmental hospitals. Initially we started with two towns & intend to offer our services to 23 villages by 2027.

**Results:**

We did face hiccups in mobilising volunteers/resources. Our strategy has minimum maintenance‐cost & high‐acceptability. Forums like AAIC‐2023 **conference** must support NGO‐activists to form workgroups with AAIC conference‐participants to further develop this concept on larger scale.

**Conclusion:**

Economical‐factors/access to therapy changes out‐come of treatment. With little training, few resources our community NGO in rural/tribal India formed well knit volunteers‐group who are giving free part‐time dedicated service. psychosocial‐support improves QOL reducing difficulties faces by resource‐poor‐southern countries. We urge participants to share views/expertise on this burning‐issue at AAIC‐2025‐venue to come out with sustain

